# A Formal Re-Description of the Cockroach *Hebardina concinna* Anchored on DNA Barcodes Confirms Wing Polymorphism and Identifies Morphological Characters for Field Identification

**DOI:** 10.1371/journal.pone.0106789

**Published:** 2014-09-18

**Authors:** Qiaoyun Yue, Keliang Wu, Deyi Qiu, Jia Hu, Dexing Liu, Xiaoya Wei, Jian Chen, Charles E. Cook

**Affiliations:** 1 Zhongshan Entry-Exit Inspection and Quarantine Bureau Technology Center, Zhongshan, Guangdong, China; 2 Guangdong Entry-Exit Inspection and Quarantine Bureau Technology Center, Guangzhou, Guangdong, China; 3 The EMBL-European Bioinformatics Institute, Wellcome Trust Genome Campus, Hinxton, Cambridge, United Kingdom; Institute of Zoology, Chinese Academy of Sciences, China

## Abstract

**Background:**

*Hebardina concinna* is a domestic pest and potential vector of pathogens throughout East and Southeast Asia, yet identification of this species has been difficult due to a lack of diagnostic morphological characters, and to uncertainty in the relationship between macroptyrous (long-winged) and brachypterous (small-winged) morphotypes. In insects male genital structures are typically species-specific and are frequently used to identify species. However, male genital structures in *H. concinna* had not previously been described, in part due to difficulty in identifying conspecifics.

**Methods/Principal Findings:**

We collected 15 putative *H. concinna* individuals, from Chinese populations, of both wing morphotypes and both sexes and then generated mitochondrial COI (the standard barcode region) and COII sequences from five of these individuals. These confirmed that both morphotypes of both sexes are the same species. We then dissected male genitalia and compared genital structures from macropterous and brachypterous individuals, which we showed to be identical, and present here for the first time a detailed description of *H. concinna* male genital structures. We also present a complete re-description of the morphological characters of this species, including both wing morphs.

**Conclusions/Significance:**

This work describes a practical application of DNA barcoding to confirm that putatively polymorphic insects are conspecific and then to identify species-specific characters that can be used in the field to identify individuals and to obviate the delay and cost of returning samples to a laboratory for DNA sequencing.

## Introduction

The Blattaria (cockroaches) are a diverse order of some 4000–4,500 species, the majority of them denizens of tropical forests, but about 40–50 of all the known cockroach species are important domiciliary pests or house frequenting dwellers [1], [2]. They carry numerous pathogens and could potentially transmit disease to humans [2]–[5]. *Hebardina concinna* is one of these pests. *H. concinna* is found in human dwellings and is believed to be a primary house pest [1], and could potentially transmit disease to humans; hence monitoring populations of this cockroach and identifying individuals within human dwellings is relevant for public health. However, due to wing-length plasticity within this species and related cockroaches, identification of individuals to the species level has been problematic.

The difficulty in describing *H. concinna* is reflected in the literature. De Haan first described this species as brachypterous [6] with the name of *Blatta* (*Periplaneta*) *concinna*. Shiraki later described females as brachypterous and males as macropterous [7]. Hebard erected a new genus of *Blattina* with *Blatta* (*Periplaneta*) *concinna* as type specimen and described the tegmina as moderately reduced, but without providing detailed descriptions of the tegmina or of male genital structures [8], although later work showed that the generic name of *Blattina* was preoccupied by Germar in 1842 [9]. Bey-Bienko replaced *Blattina* Hebard with *Hebardina* in order to memorialize Hebard and separated *Hebardina* Bey-Bienko from *Periplaneta* Burmeister on the basis of shortened elytra and hind wings [10], while Bruijning showed that the length of tegmina and the hind-wings of *H. concinna* varied widely, and listed all the tegminal lengths of the specimens that he examined [11]. Asahina then described macropterous specimens from Thailand [1], and most recently Roth examined *H. concinna* specimens collected from Krakatau, Sumatra, Java, India, and the Philippine Islands and noted extensive wing polymorphism [12].

In sum, most authors have focused on using wing morphology to describe this species but this is plastic in many cockroaches, including–as we show below–in *H. concinna*, and therefore of poor utility for species identification. Wing polymorphism exists in many insect orders, and individuals of the same species can be brachypterous or macropterous. In some insects these differences are sex-based (termed dimorphism), but in many species, including *H. concinna,* these differences occur in both males and females and are a response to varying environmental factors during nymphal stages, such as photoperiod, temperature, nutritional status, or population density. In general, good environmental conditions allow high populations whose individuals develop large wings and disperse [13].

To date wing polymorphism in Blattodea has rarely been studied, and is made difficult by their cryptic, nocturnal habits [14] and by the difficulties in identifying species by morphological examination [15]–[18]. In addition, identification based on morphology has some limitations, for instance it is difficult, even for specialists, to accurately identify females and immature stages [19].

DNA barcoding was developed by Paul Hebert and colleagues in 2003 [20], [21], and in the decade since this method has become an important tool for the identification of insects, including cockroaches [22]–[24]. “Integrated taxonomy” [25], [26] or more specifically “Barcodes and morphology taxonomy” or just “B&M taxonomy” [27] combines the power of DNA barcoding with traditional taxonomic methods and the authors above, and we ourselves, believe it should be integrated into the research framework. Nevertheless, many barcoding studies are not integrated with taxonomic research [28], [29]. Here we present an integrated barcoding and morphological study of a cockroach.

There is a broad consensus that barcodes contain information relevant for species delimitation, although in some cases a single mitochondrial marker is insufficient as a sole criterion [30]. Notably, successful barcoding identification depends upon genetic diversity being markedly lower within than between species [31]. The commonly adopted standard 658 bp COI segment has proven to be highly informative and useful for species-level identification [27], [32], including the matching of morphotypes in species with polymorphic forms. Despite increasing use of DNA-based methods morphology remains the most commonly used method in taxonomic research despite suggestions to abolish it altogether [33], although the future role of morphology in the age of genomes is anyone’s guess [27]. The integrated B&M taxonomic method is potentially a very fruitful one [27], and our study proves its utility.

We describe, for the first time, a detailed morphology for male genital structures in *H. concinna*, compare the male genital structures of both macropterous and brachypterous specimens carefully, and compare these morphological observations with DNA barcode sequences (mitochondrial COI and COII) from males and females of *H. concinna* and related species. We also provide a complete morphological re-description of this species based on samples collected in China.

## Materials and Methods

### *H. concinna* specimens

Nine putative males and six females of *H. concinna*, as well as reference individuals from other blattotid species, were collected with a sweep net at night in the leaves and litter layer in woody habitats with the assistance of headlight. Specific permission was not required for collecting in these localities, and the GPS coordinates (latitude and longitude) were provided in Tables 1 and 2. No endangered or protected species were collected for this work.

**Table 1:** *Hebardina concinna* specimens collected for morphological study.

Tegmena type	Sex	Collectionlocalitywithin Guangdong	Collectionlocation (N/E)	Collectiondate	Collector	Specimenaccessionnumber	Pronotumlength(mm)	Tegmenlength(mm)	Bodylength(mm)
Macropterous	♂	Huizhou	23°11′10.80″/114°30′41.36″	12-Oct-2010	HUANG YW	HC-16a*	4.5	15.7	16.9
	♂	Huizhou	23°11′10.80″/114°30′41.36″	06-May-2012	HUANG YW	HC-16b	4.8	15.8	16.2
	♂	Zhongshan	22°31′10.15″/113°25′02.10″	06-Apr-2013	Liu DX	HC-126a*	4.7	14.5	20.4
	♂	Zhongshan	22°30′15.95″/113°24′37.36″	02-Apr-2013	YUE QY	HC-126b	4.3	15.8	15.3
	♂	Zhongshan	22°31′10.15″/113°25′02.10″	06-Apr-2013	WU KL	HC-126C	4.5	14.5	15.4
	♀	Zhongshan	22°31′10.15″/113°25′02.10″	22-Oct-2012	YUE QY	HC-188a*	4.3	15.5	15.4
	♀	Zhongshan	22°31′10.15″/113°25′02.10″	06-Apr-2013	WU KL	HC-188b	5.3	16.9	21.2
	♀	Zhongshan	22°31′10.15″/113°25′02.10″	06-Apr-2013	WU KL	HC-188C	4.5	14.4	15.1
Brachypterous	♂	Zhongshan	22°31′10.15″/113°25′02.10″	06-Apr-2013	WU KL	HC-305a	4.9	10.1	15.4
	♂	Zhongshan	22°31′10.15″/113°25′02.10″	06-Apr-2013	Liu DX	HC-305b*	4.7	9.9	14.5
	♂	Zhongshan	22°31′10.15″/113°25′02.10″	06-Apr-2013	WU KL	HC-305c	4.9	10.7	16.9
	♂	Zhongshan	22°56′31.51″/112°03′38.32″	06-Apr-2013	WU KL	HC-305d	4.3	9.9	15.1
	♀	Zhongshan	22°31′10.15″/113°25′02.10″	22-Oct-2012	YUE QY	HC-187a*	5.3	10.6	14.9
	♀	Zhongshan	23°11′10.80″/114°30′41.36″	22-Oct-2012	YUE QY	HC-187b	5.1	10.4	15.8
	♀	Zhongshan	23°11′10.80″/114°30′41.36″	06-Apr-2013	Liu DX	HC-187c	4.7	10.2	16.5

Macropterous is defined as tegmen fully developed and extending well beyond the end of abdomen; brachypterous is defined as tegmen just about reaching to the hind margin of the second abdominal tergum. Note that there is no overlap in tegmen length between the two size classes. Individuals marked with an asterisk (*) were selected for DNA barcoding.

**Table 2:** Sequences used for DNA Barcode analysis.

Family	Species	Individualdescriptors(H. concinna only)	Accession No.for COI	Accession No.for COII	Collection location (N/E)
Blattidae	*Hebardina* *concinna*	Macropterous ♂ HC6a	**KF640073**	**KF876003**	23°11′10.80″/114°30′41.36″
		Macropterous ♂ HC126a	**KF640074**	**KF876004**	22°31′10.15″/113°25′02.10″
		Macropterous ♀ HC188a	**KF640076**	**KF876006**	22°31′10.15″/113°25′02.10″
		Brachypterous ♂ HC305b	**KF640077**	**KF876007**	22°31′10.15″/113°25′02.10″
		Brachypterous ♀ HC187a	**KF640075**	**KF876005**	22°31′10.15″/113°25′02.10″
	*Periplaneta americana*		KC617846		
			**KF640070**		22°34′14.10″/113°32′06.94″
			JQ350707		
			JN900479		
				DQ181546	
				EF363225	
				M83971	
	*Periplaneta australasiae*		**KF640069**		22°34′43.17″/113°26′17.78″
			AM114928		
	*Periplaneta* *fuliginosa*		JQ350729		
			AB126004	AB126004	
				AB014067	
				JN615391	
				DQ874312	
Ectobiidae	*Blattella* *germanica*		JQ267496		
			**KF64007**1		22°29′39.54″/113°24′32.46″
			**KF640072**		22°29′49.54″/113°25′02.10″
				DQ874268	
				EF363216	
				FJ806874	
	*Blattella* *bisignata*		NC018549	NC018549	
			JX233805	JX233805	
Blaberidae	*Rhabdoblatta* *atra*		**KF640066**	**KF876000**	22°09′43.36″/108°27′34.36″
	*Rhabdoblatta bielawskii*		**KF640067**	**KF876001**	22°08′59.45″/108°11′50.38″
	*Rhabdoblatta marginata*		**KF640068**	**KF876002**	22°12′38.17″/108°09′19.28″

Sequences with accession numbers starting with KF (in bold) are newly reported in this paper, and collection locations are reported. Other sequences were retrieved from NCBI GenBank: for collection locations see the GenBank accession. COI and COII accession numbers in the same row indicate sequences from the same individual: otherwise only a COI or a COII sequence was available for that individual. Note that within the Blattidae COI and COII sequences from the same individual are available only for individuals newly reported in this paper, from a single *P. fuliginosa,* and from a single *B. bisignata*: a combined COI/COII dataset with only these 10 individuals from within the Blattidae would not have been useful for analyses aimed at determining the relationship between *H. concinna* and other Blattidae, so separate COI and COII datasets were compiled and analyzed independently.

### Morphological study

#### General morphology

Our terminology follows McKittrick 1964 [34], Grandcolas 1996 [35], Anisyutkin 2010 [36] and Anisyutkin 2013 [37]. The genital segments of the examined specimens were macerated in 10% KOH and observed in glycerin with a Zeiss Discovery V12 stereomicroscope. Wings were floated in hot water until fully spread, embedded in neutral balsam, then mounted on slides and covered with coverslips. Drawings were made using a Zeiss Discovery V12 stereomicroscope fitted with a Canon PowerShot G1X digital camera and drawn using Adobe Illustrator CS6. All images of specimens were photographed using a Canon 60D plus a Canon EF 100 mm f/2.8L IS USM Macro lens combined with Helicon Focus software. All specimens studied were pinned in a natural posture and deposited in the medical vector collections of the Zhongshan Entry-Exit Inspection and Quarantine Bureau (ZSCIQ). The specimens we collected in China fully match other published morphological descriptions of *H. concinna* individuals from other geographic locations [6], [38], [39].

#### Quantitative morphology

**Figure 1:**
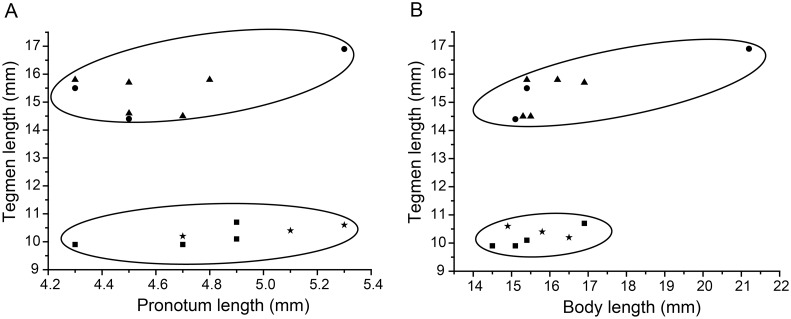
Dotplots of Tegmen length vs Pronotum length and body length in *Hebardina concinna*. These correlated characters form statistically non-overlapping groups in macropterous vs. brachypterous individuals. A. Tegmen length vs pronotum length; B. Tegmen length vs body length. Remarks: Triangle for male macropterous; circle for female macropterous; box for male brachypterous; star for female brachypterous.

Length measurements were taken from the specimens using vernier calipers. Three measurements were taken for each of the 15 specimens: tegmen length, pronotum length, and body length (excluding tegmen length). These are reported in Table 1 and plotted (tegmen vs. pronotum and tegmen vs. body length) in Figure 1. The plots suggested that these three measures do not vary independently, and their relationships were tested using a means-independent T-test (SPSS 19.0) on pronotum/tegmen length and body length/tegment length between sexes and within morphotypes.

### Molecular methods

#### Sampled specimens

The sampled individuals were preserved in 8 mL 95% ethanol immediately after capture, this was replaced with fresh 95% ethanol twice the next day. Macropterous and brachypterous males and females were used for genomic DNA purification. Various species of *Periplaneta*, *Blattella* and *Rhabdoblatta* were also studied as references. Sampled species are summarized in Table 2.

#### Genomic DNA extraction

A single hind tibia and tarsus were removed from each specimen for DNA extraction. All instruments used to remove leg tissues were cleaned with 70% ethanol and flame sterilized between each specimen. Genomic DNA was purified with a TIANamp Genomic DNA Kit (DP304, TIANGEN). Voucher specimens were labeled uniquely and deposited in the Medical vector collections of the Zhongshan Entry-Exit Inspection and Quarantine Bureau.

#### PCR amplification

We amplified a 658 bp segment of the mitochondrial COI gene using the standard arthropod DNA barcoding primers [40] and a 601 bp segment across the mitochondrial COI and COII genes, also using previously published and widely used primers [41] from five of the 15 collected *H. concinna*. Five macropterous and brachypterous male and female individuals were selected for DNA barcoding (Table 1). COI primers were LCO1490 (GGTCAACAAATCATAAAGATATTGG) and HCO2198 (TAAACTTCAGGGTGACCAAAAAATCA). The COII primers were CO1DL (CCWCGWCGWTAYTCWGAYTAYCCWGA) and CO2DL (WGAATARRCATAWSWTCARTATCATTG).

Reaction volumes were 10× Taq polymerase buffer 5 µL, dNTP (2.5 mM each) 2 µL, primer (20 uM) 1 µL each, Taq polymerase (5 U/µL) 0.5 µL, DNA template 70–100 ng, and ddH_2_O to a total volume of 50 µL.

Reaction conditions were 95°C 3 min; 95°C 45 s, 50°C 45 s, 72°C 1 min, 34 cycles; 72°C 10 min. PCR products are stored at −20°C at the Zhongshan Entry-Exit Inspection and Quarantine Bureau.

#### PCR product purification and sequencing

PCR products were purified with a TIANgel Midi purification Kit (DP209-02), linked to a T-vector with the TIANgen pGEM-T ligation kit, at 16°C overnight, then transformed into DH5α competent cells (TakaRa Biotechnology (Dalian) Co., Ltd.) for white/blue selection. White clones on the LB-agar selection medium plate with Ampicillin (100 µg/mL), IPTG (1 mM) and X-Gal (20 µg/mL) were selected for PCR Screening with a TIANgen pGEM-T recombinant colony identification Kit, then 3–5 randomly chosen positive colonies were cultured in LB medium with Ampicillin (100 µg/mL) at 37°C overnight, plasmid DNA was purified with TIANprep Mini Plasmid Kit and then sequenced commercially (Life Technologies Corporation). Plasmid DNA is stored at −20°C, and colonies in 20% glycerol at −80°C. Mutation rates are higher in sequencing directly from PCR products than sequencing colonies from a cloned PCR product sequencing [42], [43], so we chose to sequence clones for this work. For each PCR product 3–5 sequenced clones were used for analysis: the maximum differences among different clones were 3 bp out of the 658 (0.46%). This is much lower than the species limitation proposed by Hebert in 2003 [21], so a consensus sequence for each clone was used for all analyses.

#### Phylogenetic analysis

Sequences from H. concinna and other species were submitted to the International Nucleotide Sequence Database Collaboration via NCBI GenBank. As noted above we used two primer sets: one amplified a section of the mitochondrial COI gene and a second amplified a short stretch of the 3′ terminus of the COI gene, the tRNA-leu gene, and the 5′ 306 bp of the COII gene. For this second amplicon the tRNA-leu and 3′ COI sequences have little variation, and we used only the COII sequences from this amplicon for our analysis.

We also identified nine additional cockroaches in GenBank for which either or both the COI and COII regions were available and created separate COI and COII data sets for phylogenetic analysis (Table 2). Unfortunately there were not enough individuals for which both regions had been sequenced to assemble a combined dataset using both genes so we analyzed the two data sets separately.

We estimated maximum likelihood and neighbor-joining phylogentic trees for both the COI and COII data sets using Mega 5.2 [44], and tested robustness of the results using non-parametric bootstrapping. For both data sets the most appropriate maximum likelihood model (TN+I) was identified using the model testing function of Mega and this model was used to estimate the ML tree for each data set. Support for each branch was assessed using the same model for 1000 bootstrap replicates. Neighbor joining trees were constructed for each data set using the same Tamura-Nei model and also tested using 1000 non-parametric bootstrap replicates.

## Results

### Taxonomic description

***Hebardina*** **Bey-Bienko, 1938** [10] **.**

*Blattina* Hebard, 1929: 84 [8].

*Hebardina* Bey-Bienko, 1938: 234 [10].

Type species: *Blatta* (*Periplaneta*) *concinna* de Haan, 1842 [6].

### Generic diagnosis

Middle sized and uniformly dark colored cockroaches. Sexual dimorphism inconspicuous. Tegmen and wings fully developed or reduced. Front femur Type A. Tarsus with 2 rows of spines along lower margin; pulvilli and arolium present; post-tarsus claws symmetrical, unspecialized. First abdominal tergum of male specialized, with a densely setose medial tergal gland. Supra-anal plate and paraprocts symmetrical. Hypandrium slightly asymmetrical. Left phallomere with L2d large, occupied upper margin. L3d with a hook in the terminal. L2v elongated, plate-like and additional with a spiniform curved process. Right phallomer with caudal part of sclerite R1 plate-like, processes; sclerite R2 with groove at cranial part and right side; R4 palte-like and inset in the groove of sclerite R2.

### This species has also been described with the following Names [9]

***Hebardina concinna*** **(de Haan, 1842).**

*Blatta* (*Periplaneta*) *concinna* Haan, 1842: 50.

*Periplaneta borrei* Saussure, 1873: 113. Synonymized by Princis, 1966: 467.

*Stylopyga concinna*, Krauss, 1902: 747.

*Methana concinna*, Kirby, 1904: 136.

*Stylopyga concinna*, Shiraki, 1906: 17, 30.

*Blatta concinna*, Shelford, 1910: 15.

*Blattina concinna*, Hebard, 1929: 12, 84.

*Blatta concinna*, Hanitsch, 1932: 5.

*Blattina concinna*, Chopard, 1934: 728.

*Hebardina concinna*, Bey-Bienko, 1938: 234.

*Blatta concinna*, Dammerman, 1948: 484, 555.

*Blattina concinna*, Bruijning, 1948: 39, 114.

*Hebardina concinna*, Princis, 1950: 204, 210.

### General Description

Sizes of the examined *H. concinna* specimens are summarized in Table 1.

**Figure 2:**
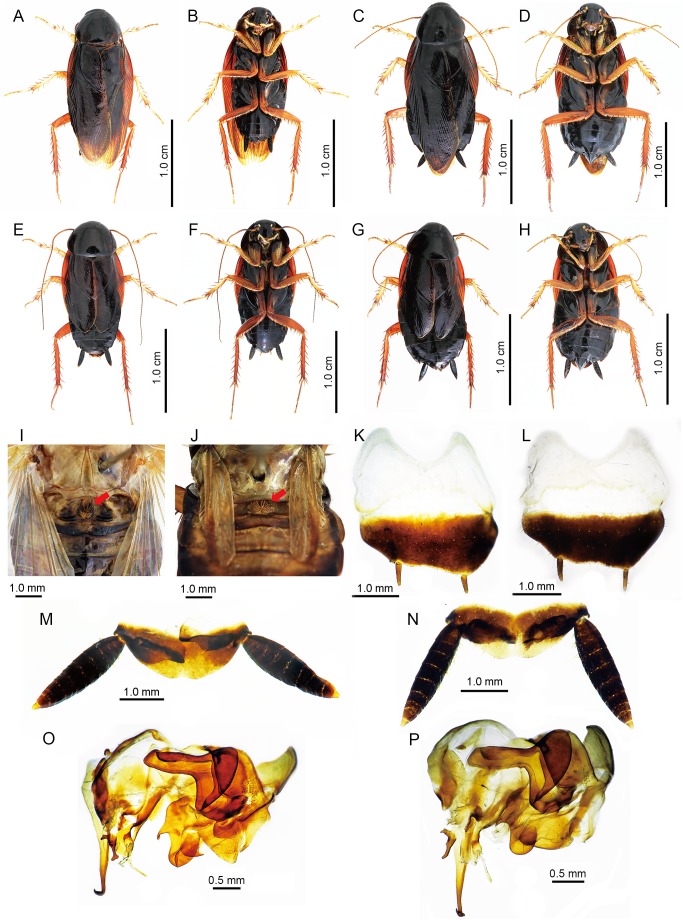
Overview of *Hebardina concinna* morphology clearly showing differences in wing length. A–D. Macropterous. A.♂, dorsal view. B. ♂, ventral view. C. ♀, dorsal view. D. ♀, ventral view. E–H Brachypterous. E. ♂, dorsal view. F. ♂, ventral view. G. ♀, dorsal view. H. ♀, ventral view. I–J. First abdominal tergum. I. ♂, macropterous. J. ♂, brachypterous. K–L. hypandrium, dorsal view. K. ♂, macropterous. L. ♂, brachypterous. M–N. supra-anal plate, ventral view. M. ♂, macropterous. N. ♂, brachypterous. O–P. male genitalia, dorsal view. O. macropterous, P. brachypterous. Remarks: Arrow (←) indicate the medial tergal gland on the first abdominal tergum.

**Figure 3:**
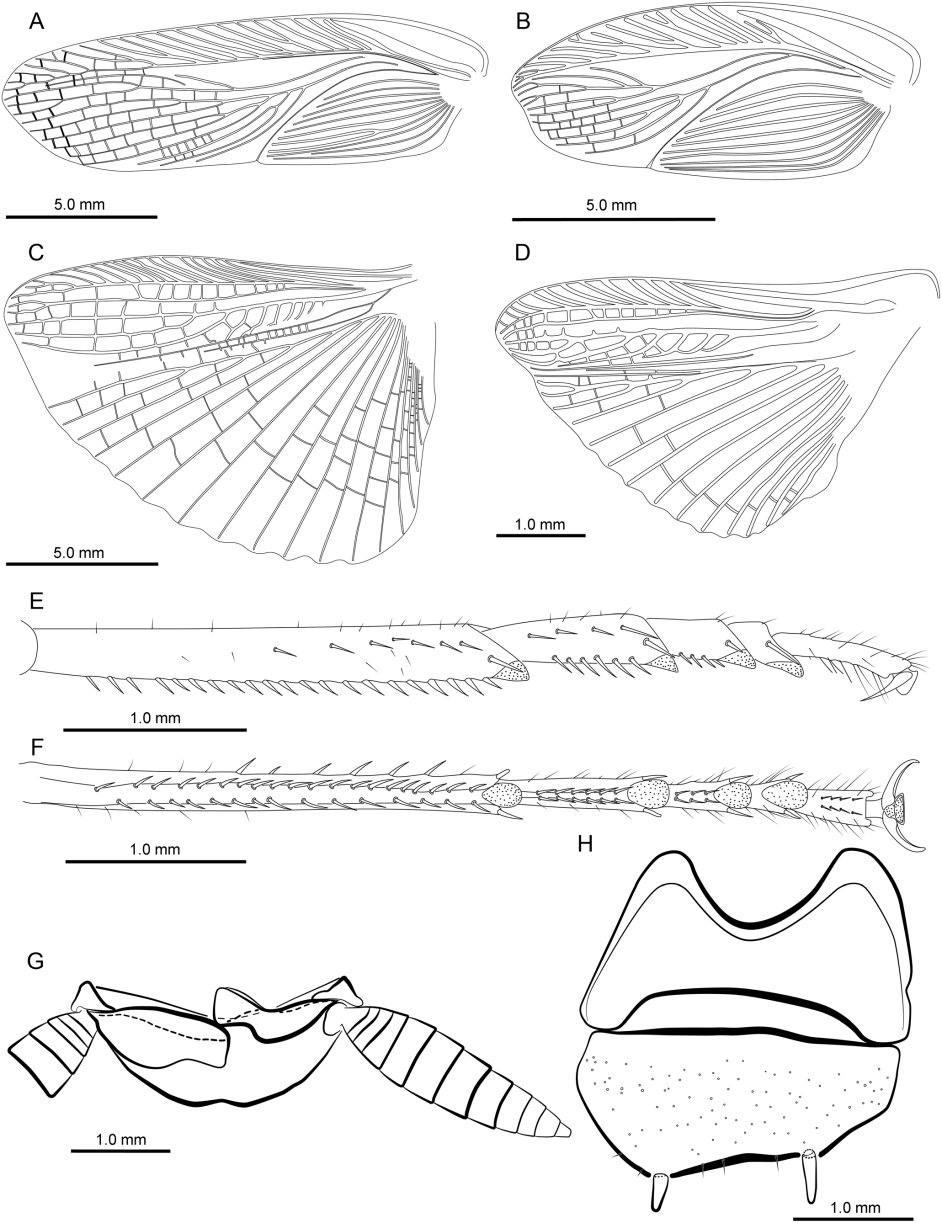
*Hebardina cocinna*: primary morphological characters and macropterous vs. brachypterous wings and tegmina. Wings and tegmina differ in macropterous and brachypterous *Hebardina concinna* whereas tarsi and abdominal morphology do not. A. macropterous, tegmen. B. brachypterous, tegmen. C. macropterous, wings. D. brachypterous, wings. E. hind tarsus from outside. F. hind tarsus from below. G. supra-anal plate, ventral view. H. hypandrium, ventral view.

**Figure 4:**
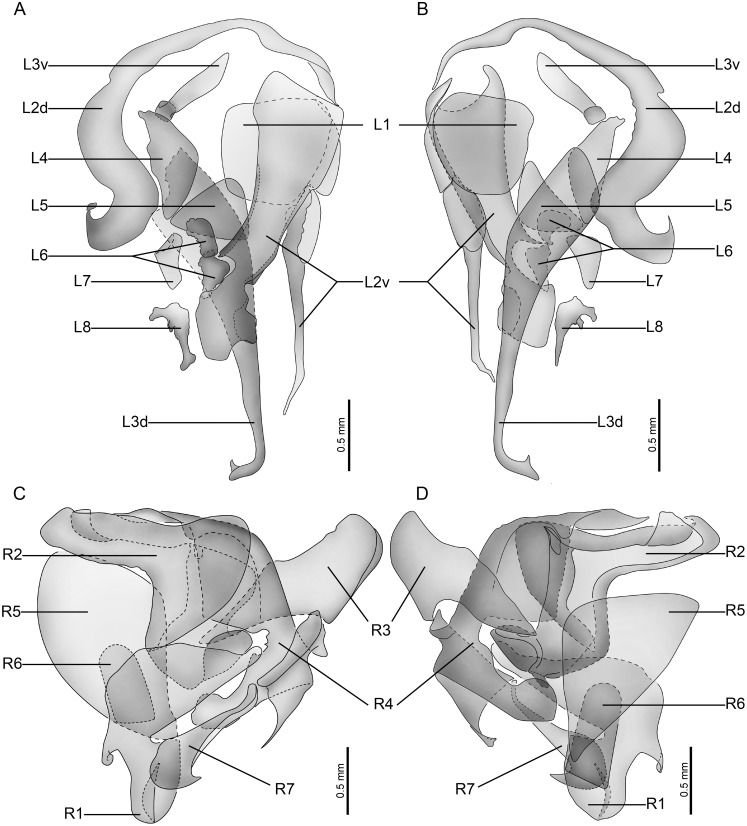
Male genital structures of *Hebardina concinna*. A. left phallomere, dorsal view. B. left phallomere, ventral view. C. right phallomere, dorsal view. D. right phallomere, ventral view. (R for right, L for left, v for ventral, d for dorsal).

Except for the differences in the length and the vein of the tegmen and hindwings, the morphology and structure of the male genitalia of the macropterous and brachypterous specimens are identical as illustrated in Figure 2, Figure 3 and Figure 4.

Body reddish brown (Figs. 1. A–H). Head reddish brown (Figs. 1. B, D, F, H). Antennae yellowish brown (Figs. 1. A–H). Eyes black (Figs. 1. B, D, F, H). Ocelli yellowish (Figs. 1. B, D, F, H). Maxillary palpi yellowish (Figs. 1. B, D, F, H). Coxa reddish brown, and other leg segments yellowish brown (Figs. 1. B, D, F, H). Vertex with interocular space equal to the distance between antennal sockets (Figs. 1. B, D, F, H). Third, fourth, and fifth maxillary palp the same length (Figs. 1. B, D, F, H). Front femur Type A2 (Figs. 1. B, D, F, H). All tarsi with 2 rows of spines along lower margin; pulvilli and arolium present; post-tarsus claws symmetrical, unspecialized (Figs. 2. E–F). The tegmina and wings in this species occur in two types, macropterous and brachypterous (Figs. 1. A–H; 2. A–D). The former is fully developed extending well beyond the end of abdomen (Figs. 1. A–D). And the later is smaller, just about reaching to the hind margin of the second abdominal tergum (Figs. 1. E–H). Tegmen with subcostal veins strongly oblique and without branches. The branches of the radiusal veins and cubital veins of macropterous individuals are more numerous than in the brachypterous individuals (Figs. 2. A–B). The hind wing of macropterous individuals has thickened costal veins, particularly in the apical area. Medial vein simple, without branches or with one branch. Cubital vein with 3 complete branches to the apex and some branches behind them not reaching the apex, triangular apical area moderate in size. Hind wing of brachypterous individuals reduced and smaller than in macropterous individuals, and without triangular apical area (Figs. 2. C–D).

First abdominal tergum of male specialized, with a densely setose medial tergal gland (Figs. 1. I–J). Male genitalia with supra-anal plate symmetrical, hind margin convex with a weak medial indentation, paraprocts nearly symmetrical. Cercus coned (Figs. 1. M–N; 2. G). Hind margin of hypandrium slight asymmetrical, left side slightly more convex than right side; styli small, cylindroid (Figs. 1. K–L; 2. H). Left phallomere with L2d large, caudal slightly widened and with hooklike terminus, cranial section bent; Elongated L3d occupying ventral part of phallomere, with a hook in the terminus; Sclerite L1 simple, large, plate-like, slightly squared; Small L3v elongated, with one side enlarged, the other side plated; L2v large, occupying dorsal and right side of phallomere, elongated, plate-like and additionally with a spiniform curved process; Sclerite L4, L5, L7 triangular, weakly sclerotized, situated on middle part of phallomere; Sclerite L6 elongated, middle part strongly sclerotized; Sclerite L8 small, with three round apices, located at the left side of L3d. (Figs. 1. O–P; 3. A–B). Right phallomere sclerite R1 trilateral, apex round and with a round small process on the left basal part; sclerite R2 is in the shape of a numeral "7″ with a groove at the cranial end on the right, there is an acute process in the corner of sclerite R2; the upper part of sclerite R4 widened, inset in the groove of sclerite R2, cranial margin slightly curly and with a rounded apophysis in lower margin, connected with the acute process at the corner of sclerite R2, basal part bifurcate, branch sheeted; sclerite R3, R5, R6 sheeted, situated on right side and left side of phallomere; sclerite R7 sheeted, left side widened and rounded, with a pointed apex in lower margin, right side slender. (Figs. 1. O–P; 3. C–D).

Male genital structures of the macropterous and brachypterous specimens are the same.

### Quantitative morphology

Tegmen length was significantly different between the macropterous and brachypterous *H. concinna* morphotypes (t-test, *P*<<0.01): tegmen lengths of the macropterous individuals were greater than the brachypterous ones without any overlap. Moreover, the ratios of pronotum/tegmen length and body/tegmen length were significantly different (t-test, *P*<<0.01, Table 3). This relationship is visually apparent in Figure 1, where macropterous individuals and brachyperous individuals cluster separately when tegmen length is plotted against body length or pronotum length. There were no significant differences between the sexes in any characters (Table 3). Tegmen length, tegmen/pronotum length, and tegmen/body length were significantly different between the macropterous and brachypterous *H. concinna* morphotypes, but were not significantly different between males and females within the same morphotype (Table 3).

**Table 3:** Results of t-tests for quantitative morphology.

Morphtype	No. of individuals(males+females)	Items	P value	Significance
M & B	15 (8+7)	TL	**7.46E-08<<0.01**	**significantly** **different**
		PL/TL	**9.18E-09<<0.01**	**significantly** **different**
		BL/TL	**4.41E-08<<0.01**	**significantly** **different**
		PL	0.193>0.5	Insignificant
		BL	0.36>0.5	Insignificant
Mm & Mf	8 (5+3)	TL	0.631>0.5	Insignificant
		PL/TL	0.29>0.5	Insignificant
		BL/TL	0.56>0.5	Insignificant
		PL	0.598>0.5	Insignificant
		BL	0.562>0.5	Insignificant
Bm & Bf	7 (4+3)	TL	0.352>0.5	Insignificant
		PL/TL	0.297>0.5	Insignificant
		BL/TL	0.924>0.5	Insignificant
		PL	0.195>0.5	Insignificant
		BL	0.734>0.5	Insignificant

There Are Clear and Significant Correlations between Tegmen Length (TL), Pronotum Length (PL), and Body Length (BL). M, Macropterous; B, Brachypterous; Mm, Macropterous Male; Mf, Macropterous Female; Bm, Brachypterous Male; Bf, Brachypterous Female.

### Distribution

China (Fujian, Guangdong, Guangxi, Hainan, Yunnan, Guizhou, Sichuan, Xizang, Beijing); Vietnam; Thailand; Burma; Malaysia; Indonesia; Australia; Japan [38], [45].

### DNA barcoding

#### PCR products of H. concinna

Amplified COI sequences (not including primers) for all individuals were 658 bp, with no stop codons, insertions or deletions, and could be translated into 219 amino acids without any interruption, mean nucleotide content of COI sequences was A (31.9%), T (37.4%), G (15.7%) and C (15.0%). As reported for other insect mitochondrial sequences [46]–[48], A + T (69.4%) was in higher proportion than G + C (30.7%), and were comparable to those typical of insects in general for this COI gene region [46]. The 601 bp COII amplicon (after removing primer sequences) included 199 bp of the 3′ terminus of the COI gene, the entire 96 bp tRNA-Leu gene, and the 306 5′ bases of the COII gene. The mean nucleotide content of this amplicon was A (40.4%), T (36.0%), G (10.5%) and C (13.1%), again A + T (76.4%) was in much higher proportion than G + C (23.6%), as is usual for insects.

#### Phylogenetic analysis

**Figure 5:**
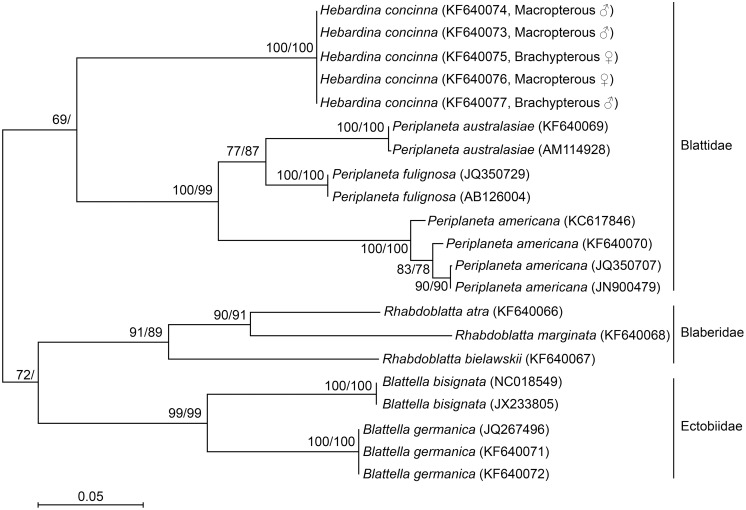
Distance matrix/neighbor joining phylogenetic tree based on 658 bp of aligned cockroach COI nucleotide sequences. The maximum likelihood tree was topologically identical, although with differing branch lengths. Numbers on branches represent support from 1000 non-parametric bootstrap replicates for distance matrix-NJ analysis and maximum likelihood analysis, respectively. Missing numbers indicate branches with less than 50% support. This analysis clearly supports grouping of the five *H. concina* individuals as a single species.

**Figure 6:**
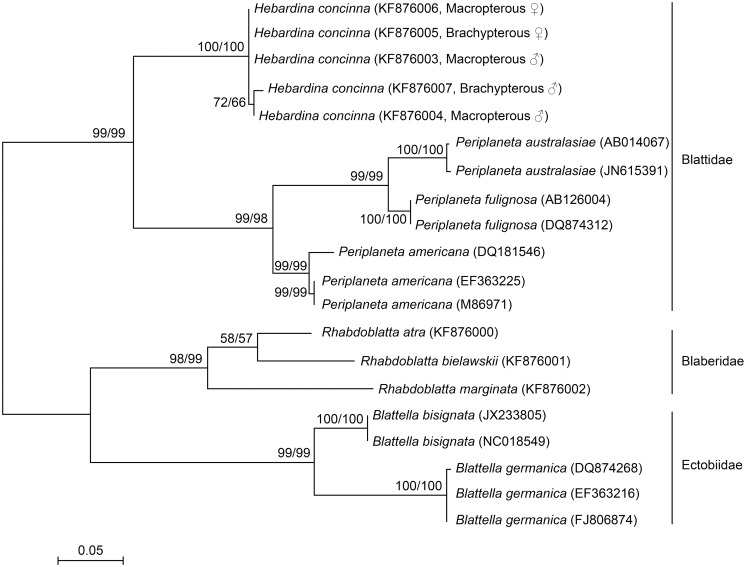
Distance matrix/neighbor joining phylogenetic tree based on 306 bp of aligned cockroach COII nucleotide sequences. The maximum likelihood tree was topologically identical, although with differing branch lengths. Numbers on branches represent support from 1000 non-parametric bootstrap replicates for distance matrix-NJ analysis and maximum likelihood analysis, respectively. Missing numbers indicate branches with less than 50% support. This analysis clearly supports grouping of the five *H. concina* individuals as a single species.

Sequences of 30 individuals belonging to 9 species, 4 genera, and 3 families were analyzed. Phylogenetic trees were estimated using aligned COI or COII data sets as described in the methods and presented in Figures 5 and 6. The COI data set included 21 taxa with 658 nucleotides. Of the 658 nucleotides 41 of 219 first positions, 5 of 219 second positions, and 170 of 220 third positions were variable, but this variation resulted in only 14 of 220 amino acid changes. Two of the variable sites at first positions and 12 at third positions occurred only in one taxon. The COII data set included 21 taxa with 306 nucleotide. Of the 306 nucleotides 33 of 102 first positions, 12 of 102 second positions, and 67 of 102 third positions were variable, with 28 of 102 amino acids varying. Three of the variable sites at first positions and ten at third positions occurred only in one taxon.

The aim of the phylogenetic analysis was to assess the relatedness of macropterous and brachypterous *H. concinna* individuals. The short mitochondrial sequences used for DNA barcoding are appropriate for this purpose. For deeper phylogenetic analyses such short mitochondrial DNA barcoding region(s) may be uninformative. Nevertheless, the data sets we have assembled do strongly support the three cockroach families represented: the Blattidae, Blaberidae, and Ectobiidae. This result is completely concordant with the morphological classification. Within each family each named genus; *Hebardina*, *Periplaneta*, *Rhabdoblatta*, and *Blattella* are also supported by at least 90% of bootstrap replicates.

The five *H. concinna* individuals shared identical COI sequences and differed only by one or two bases in the COII region: this phylogeny shows that these individuals are clearly distinct from other species within the Blattidae. There is no genetic distinction between macropterous and brachypterous individuals in either sex. This agrees with our observation that male genital morphology is identical in cockroaches with both wing morphs and we conclude, as hypothesized, that macroptery and brachyptery in both males and females of *H. concinna* are different ecotypes of the same species.

## Discussion

Wing polymorphism exists in many insects, including the Coleoptera, Diptera, Hemiptera, Hymenoptera, Orthoptera, Lepidoptera, and Thysanoptera. The reasons for this polymorphism may relate to both population size and selection pressure. For instance, when population densities are high the proportion of macropterous brown plant hoppers *Nilaparvata lugens* is higher than when it is low [49]. This same phenomenon has also been described in Coleoptera, Diptera, Hemiptera, Hymenoptera, Orthoptera, Lepidoptera, and Thysanoptera [50]. When populations are low, nymphs develop into solitary brachypterous adults, the energy saved by not building large wings and flight muscles is instead directed into reproduction, thus allowing populations to increase rapidly [51]. Conversely, when populations are high, individuals develop into macropterous gregarious adults that are strong flyers who disperse to other habitats with lower population densities. There is a resource allocation trade-off relationship between the development and maintenance of flight muscles and reproductive capacity. Our results clearly demonstrate wing polymorphism in cockroaches that is not sex-based, and therefore is likely to have an environmental trigger. Further research using *H. concinna* or other Blattodea might reveal these triggers in cockroaches.

Wing polymorphism is rarely reported in Blattodea. This is likely due to their secretive, nocturnal habits, to the lack of molecular data, and the lack of description of male genital morphology: these factors together mean that collecting is difficult and identification of collected individuals is problematic. Male genital structures are the most important characters used for species identification in many insects, especially for species that exhibit polymorphism in other characters. In particular, McKittrick [34] first used male genital characters to describe cockroach species. Following this work male genital characters have been widely used by many taxonomists such as Anisyutkin, Roth, and Grandcolas, to distinguish different species of Blattodea [35], [36], [39], [52]. Our results show that male genital structures are also useful for identification of *H. concinna*, but also demonstrate that it may be necessary to use other methods, such as DNA barcoding, to identify a cohort of conspecific animals in order to develop morphological keys to the genitalia.

Our results showed that, for population in China, both *H. concinna* males and females have macropterous and brachypterous morphotypes and allowed us to develop morphologically based criteria for identification of (males of) this species. This means that entomologists, and in this case pest control agents and public health officials, can be trained to identify *H. concinna* quickly in the field by picking up individuals and looking at their abdomens without the need to bring samples back to a laboratory for costly and time consuming DNA barcoding. We have no doubt that similar efforts will allow leveraging of additional up front investments in laboratory barcoding to develop field-friendly keys for morphological identification of previously problematic species across the Metazoa.

*H. concinna* is distributed throughout East and Southeast Asia, and the type location is in Malaysia. Our work is based on individuals collected in China and our results therefore remain to be confirmed for the rest of this species’ range. In particular, our work represents the only report of mitchondrial sequences from this species, so further work is necessary to understand genetic variation in “*H. concinna*” across the rest of its range. However, our experience with numerous cockroach species strongly suggests that our observations on genital morphology will be confirmed across the entire range of the species, and we hope our work will stimulate further research on this topic.
